# Measurement of Optical Rubidium Clock Frequency Spanning 65 Days

**DOI:** 10.3390/s22051982

**Published:** 2022-03-03

**Authors:** Nathan D. Lemke, Kyle W. Martin, River Beard, Benjamin K. Stuhl, Andrew J. Metcalf, John D. Elgin

**Affiliations:** 1 Department of Physics and Engineering, Bethel University, St. Paul, MN 55112, USA; lemnat@bethel.edu; 2 Blue Halo, Albuquerque, NM 87123, USA; kyle.martin@bluehalo.com (K.W.M.); river.beard@bluehalo.com (R.B.); 3 Space Vehicles Directorate, Air Force Research Laboratory, Kirtland Air Force Base, Albuquerque, NM 87117, USA; ben.stuhl@sdl.usu.edu (B.K.S.); aj.metcalf2@gmail.com (A.J.M.); 4 Space Dynamics Laboratory, North Logan, UT 84341, USA

**Keywords:** atomic clock, helium permeation, two-photon spectroscopy

## Abstract

Optical clocks are emerging as next-generation timekeeping devices with technological and scientific use cases. Simplified atomic sources such as vapor cells may offer a straightforward path to field use, but suffer from long-term frequency drifts and environmental sensitivities. Here, we measure a laboratory optical clock based on warm rubidium atoms and find low levels of drift on the month-long timescale. We observe and quantify helium contamination inside the glass vapor cell by gradually removing the helium via a vacuum apparatus. We quantify a drift rate of $4\times 10^{-15}$/day, a 10 day Allan deviation less than $5\times 10^{-15}$, and an absolute frequency of the Rb-87 two-photon clock transition of 385,284,566,371,190(1970) Hz. These results support the premise that optical vapor cell clocks will be able to meet future technology needs in navigation and communications as sensors of time and frequency.

## 1. Introduction

In recent years, optical clocks have won acclaim as some of the most precise instruments ever developed. A few examples include an optical clock comparison via fiber and free-space optical links that demonstrated agreement to 18 digits among three clocks based on different atomic species [1], and a test of fundamental symmetry between two ytterbium ion clocks at the $10^{-18}$ level [2]. Applications for transportable or even deployable optical clocks have emerged, including geodesy [3], navigation [4,5], tests of relativity [6], and communications [7]. Indeed, work is underway worldwide to produce systems that are sufficiently compact and robust to be operated outside the metrology laboratory. Some of the systems demonstrated so far utilize vapors such as molecular iodine [8] or atomic rubidium [9,10,11,12,13,14]; others utilize laser cooling and trapping of neutral atoms [3,15,16,17] or single ions [18,19]. A key enabler has been the increasing availability of optical frequency combs, which are leveraged in all optical clocks to coherently down-convert the high-frequency optical signals to the RF domain [20,21].

The choice of which atomic system to use can be seen as a trade-off between simplicity and performance. Single-ion or optical lattice clocks require multiple frequency-controlled laser systems, including one ultrastable laser for probing the very narrow clock transition, which typically requires a high-finesse reference cavity for frequency pre-stabilization. By comparison, an atomic vapor clock such as the rubidium two-photon clock described here [22,23] needs only one laser to probe the atomic sample, with no additional cooling or optical pumping, and no reference cavity. However, the performance of a warm vapor clock is limited compared to optical lattice or single-ion systems: short-term stability as characterized by the Allan deviation is typically ≥$10^{-13}$ at 1 s averaging time because of the broader atomic linewidth, and long-term stability below $10^{-14}$ remains very challenging due to thermally-activated systematic clock frequency shifts. In this work, we describe a long-term measurement of an optical rubidium clock, measuring its frequency continuously for 65 days until a laboratory power outage ended the measurement campaign. We have observed and modeled the effects of helium de-permeation from the glass vapor cell owing to the vacuum surrounding the cell, and, after removing this small effect from the frequency measurement, we find the long-term instability of the clock to be ≈$5\times 10^{-15}$ on timescales from 1 to 10 days. Finally, we measured the absolute frequency of the rubidium clock transition and found that it agrees well with prior measurements.

## 2. Materials and Methods

**Figure 1:**
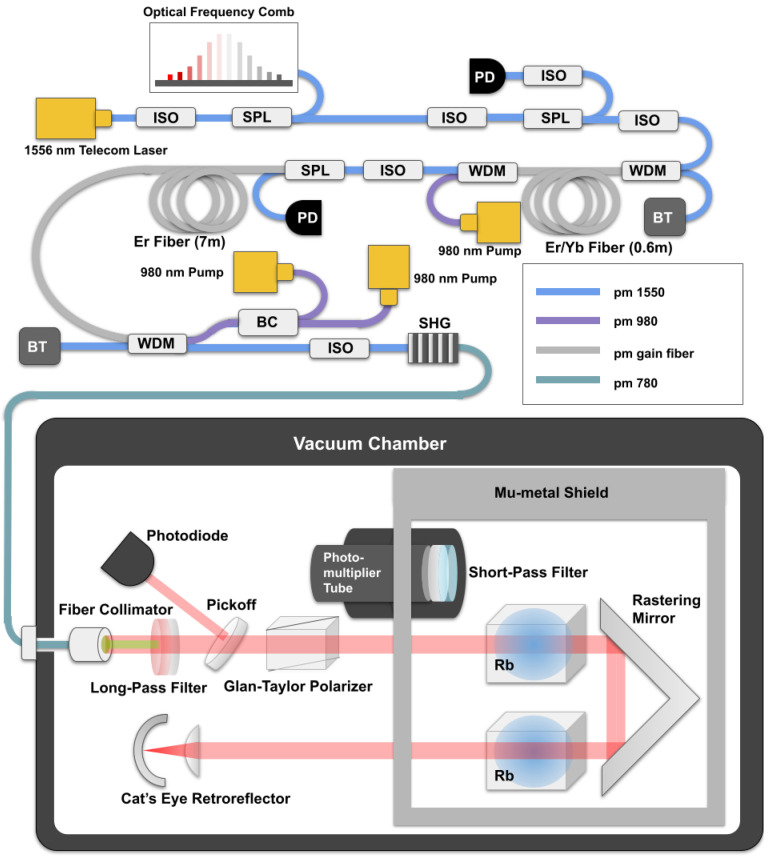
Diagram of the two-photon clock. Laser light at 1556 nm is amplified, frequency doubled, and delivered by optical fiber into a vacuum chamber via a vacuum feedthrough. From there, the light is launched into free space and encounters a filter, pickoff (for monitoring power), and polarizer before entering the vapor cells. A cat’s eye retroreflector returns the beam anti-parallel to create counter-propagating beams. A photomultiplier tube (PMT) monitors for 420 nm fluorescence from the Rb atoms. An optical frequency comb (OFC) divides the laser frequency optical frequency down to an RF signal. ISO: isolator. SPL: splitter. WDM: wavelength division multiplexer. BT: beam terminator. BC: beam coupler. PD: photodiode. SHG: second harmonic generation.

The rubidium two-photon clock utilizes the 5s ${}^{2}S_{1/2}\left(F=2\right)\to$ 5d ${}^{2}D_{5/2}\left(F=4\right)$ transition in ${}^{87}$Rb driven by two counterpropagating 778.1 nm laser beams. Counterpropagating beams eliminate the first-order Doppler shift and its associated inhomogeneous broadening. The 778.1 nm light is generated by second harmonic generation (SHG) of a commercially-available 1556 nm planar-waveguide external cavity laser [24] that has high passive frequency stability. No laser pre-stabilization to an external optical cavity is needed, since commercial laser sources are available with linewidths much narrower than 330 kHz natural linewidth of the transition. A series of optical isolators, erbium fiber amplifiers, and beamsplitters are used to deliver 1556 nm light to both a commercial erbium fiber frequency comb [25] and a ridge waveguide periodically-poled lithium niobate SHG module. The full diagram of the laser system is shown in Figure 1. Tuning and stabilization of the laser frequency are accomplished by varying the 1556 nm laser’s current and temperature. The isolators not only protect the laser from undesired optical feedback but also prevent the formation of in-fiber etalons, which are particularly detrimental to the clock stability in the presence of frequency modulation [26].

The generated 778.1 nm light is carried by optical fiber to a vacuum chamber enclosing the Rb cell apparatus. A free space laser beam is collimated and directed through a long-pass filter that passes 778 nm. Following that is a beam sampler and photodiode for monitoring the laser power and a linear polarizer. After entering a mu-metal enclosure to block surrounding magnetic fields, the beam passes through two fused silica vapor cells filled with pure rubidium-87 and heated to 100 ${}^{\circ }$C. The use of two vapor cells (length 50 mm, diameter 10 mm) effectively doubles the interaction length of the laser with the atomic vapor. A cat’s eye reflector forces the beam to retrace its path and create the counterpropagating beams.

Fluorescence from the rubidium atoms near 420 nm is collected on the photomultiplier tube (PMT). The large area PMT is positioned directly above the vapor cells for high collection efficiency. Sinusoidal frequency modulation (f=40 kHz) is applied to the laser via the injection current, and the PMT output is digitally demodulated at 3f to generate an error signal. Demodulation at 3f enables a suppression of the lock point error induced by residual amplitude modulation [27]. A proportional and integral gain loop filter stabilizes the laser frequency to the peak of the fluorescence signal. The 778.1 nm laser power is stabilized by measuring the monitor photodiode signal and feeding back to the pump current in the erbium fiber amplifier.

The erbium frequency comb is a mode-locked laser with pulse repetition rate near 160 MHz [25]. Following the mode-locked laser are two-stages of amplification and a segment of highly non-linear fiber (HNLF), generating an octave of bandwidth that permits self-referencing of the carrier-envelope offset frequency. The repetition rate of the comb is then controlled by stabilizing a beatnote between one tooth of the erbium comb with the 1556 nm laser; that optical beatnote is locked at 21 MHz. With the offset frequency stabilized at −21 MHz, the comb’s repetition rate is the two-photon clock frequency divided by an integer, and photodetection of the repetition rate accomplishes the optical-to-microwave conversion necessary to compare the two-photon clock’s output with other timing systems.

After photodetection, the repetition rate signal is amplified and sent to a Microsemi 5125A (Product names and model numbers are given for completeness. No endorsement is made by the authors. ) phase noise analyzer that is operated as a frequency counter. The reference signal is provided by the 100 MHz output of an active hydrogen maser with low phase noise. Both the hydrogen maser and the phase noise analyzer are operated inside a temperature- and humidity-controlled environmental chamber. In addition to recording the comb’s repetition rate over time, environmental signals were recorded including the lab weather (temperature, humidity, and atmospheric pressure), average PMT current, numerous out-of-loop temperature sensors on or near the vapor cell, and the drive current of the heaters stabilizing the vapor cell temperature. The hydrogen maser’s frequency is also recorded by a GPS-referenced frequency counter.

## 3. Results

Data were collected continuously for 65 days until a building power outage forced continuous data collection to cease. Below, we describe calibration of the hydrogen maser via the GPS-steered counter, modeling and accounting for the effects of helium contamination inside the vapor cell, and calculating the absolute frequency of the rubidium two-photon clock transition.

### 3.1. Calibrating the Hydrogen Maser

Hydrogen masers are susceptible to small frequency drifts and inaccuracies. Consequently, we use a GPS receiver to calibrate the hydrogen maser frequency relative to GPS, which is itself maintained by frequency standards directly realizing the SI second and is steered to track International Atomic Time (TAI). This is accomplished by counting the hydrogen maser’s 100 MHz output on a frequency counter that has both an internal time-base (Rb microwave oscillator) and accepts a reference frequency from a GPS antenna located on the roof of the laboratory. We have confirmed that any inaccuracy of this system is below $10^{-13}$ by having it directly count a second copy of the GPS reference frequency, and by calculating the Allan deviation of the counted hydrogen maser, which averaged down to $3\times 10^{-14}$ at 10 days despite the drift of the hydrogen maser.

Because the stability of our calibration measurement is lower (approximately $10^{-11}$ at 1 s and $1\times 10^{-13}$ at 1 day) than that of the hydrogen maser vs. two-photon clock measurement, we cannot directly correct for the maser frequency error point-by-point without decreasing the quality of this measurement campaign. Instead, we use the calibration for two specific purposes. First, we calculate a linear drift of $-3.2\left(4\right)\times 10^{-20}$/s (here expressed as a fractional frequency per second) in the hydrogen maser relative to GPS, averaged over the 65 day measurement, and we subtract this drift from the observed two-photon clock data. This is important for determining the two-photon clock’s drift rate, as well as the time constant associated with helium de-permeation. Second, we calculate the mean error of the hydrogen maser over the 65 day measurement ($-5.51\left(3\right)\times 10^{-12}$ in fractional frequency), and we use this correction when finding the absolute frequency of the two-photon clock below.

### 3.2. Helium Permeation

Helium is known to diffuse through glass and cause pressure-dependent atomic frequency shifts inside vapor cells [28,29,30]. To prevent helium contamination, care must be taken to remove the dissolved helium gas from the glass cell, and to reduce subsequent diffusion as much as possible through the use of helium-impermeable glasses [31,32]. An alternate and simpler approach employed here is to select a glass material with a higher permeability (in this case, fused silica) and to place the vapor cell inside a vacuum chamber; gradually, the helium will diffuse out of the cell and be removed by the vacuum pump.

The frequency measurement described here took place approximately 60 days after the vapor cell was placed under vacuum. We anticipated and observed a small, time-dependent frequency shift associated with the changing helium concentration, which we model using the time-dependent diffusion equation [28,29,33]

(1) $$\frac{\partial n}{\partial t}=D\frac{\partial^{2}n}{\partial x^{2}}$$

with *n* the helium concentration, and where the material property *D* is referred to as the diffusion constant. In the case that the vapor cell, after its construction and filling, has been exposed to helium for a sufficiently long time (several months), the initial pressure inside the vapor cell is essentially equal to the partial pressure of helium in atmosphere, approximately 4 mTorr. Likewise, the quantity of gas inside the glass can be taken to be saturated at a value of *S* times the atmospheric pressure, where the solubility *S* is also a material property. For fused silica at 100 ${}^{\circ }$C, previous measurements suggest $D\approx 3\times 10^{-7}$ cm${}^{2}$/s [34] and S≈0.02 [34], and the permeability $K=DS\approx 6\times 10^{-9}$ cm${}^{2}$/s [29]. Note that the unit cm${}^{2}$/s is equivalent to (cm${}^{3}$ at STP/cm${}^{2}$ s)/(atmosphere of pressure difference/glass thickness of one centimeter) [28].

Unlike prior work that focused on the migration of helium into a reservoir [28,29], here we seek to quantify the helium gas remaining inside the cell reservoir as it depletes over time. Along with Equation (1), we enforce the following boundary conditions. First, the helium concentration on the outer surface of the glass is zero; second, the concentration on the inside surface of the glass is equal to *S* times the vapor cell pressure *p*, which is itself slowly time-varying; third, in any time-step the quantity of gas entering the glass through its inside surface must be equal to the quantity of gas leaving the cell reservoir. We have modeled these equations and performed a finite difference simulation. We observe that, initially, *p* changes very little, as the gradient in concentration between the cell reservoir and the interior of the glass is small, whereas the pressure inside the glass changes more rapidly given the vacuum environment surrounding it. After a sufficiently long time, the concentration inside the glass reaches a steady-state distribution, and the depletion of the cell pressure is observed as an exponential decay.

For a cylindrical geometry (height *h*, outer radius *a*, inner radius *b*, R=a/b), we find

(2) $$\frac{dp}{dt}=\frac{2\pi hK}{V\ln R}p$$

with V=2πbh the interior volume of the cell. The solution of the above differential equation is an exponential decay with time constant $T_{\mathrm{theory}}=V\ln R/\left(2\pi hK\right)$. For our cell geometry, h=50 mm, a=10 mm, b=8 mm, and we expect $T_{\mathrm{cell}}\approx 34$ days. The uncertainty in the diffusion constant, solubility, and permeability is estimated to be 10% [29,34].

**Figure 2:**
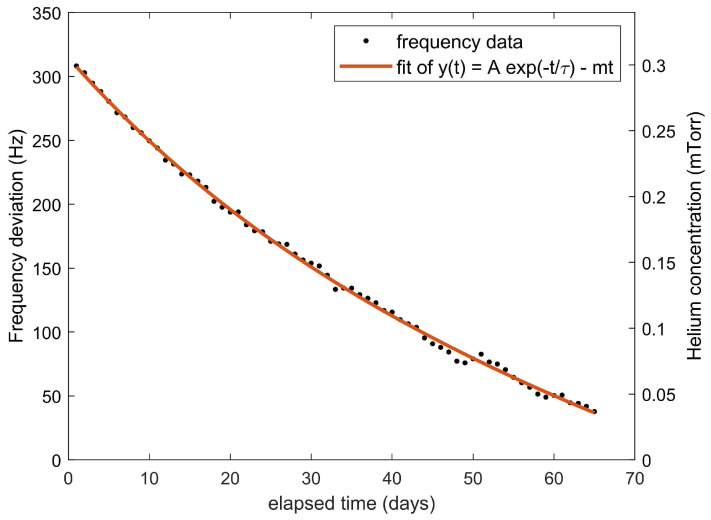
Daily average frequency data over time (dots), along with an exponential decay fitting function (red line) that also includes a small linear drift. The data and fit are manually offset so that they gradually approach y = 0, which corresponds to a helium-free condition. The observed time constant $T_{\mathrm{cell}}$ is 38(10) days. The right y axis displays the inferred remaining partial pressure of helium in mTorr.

To search for a comparable effect in the two-photon clock frequency data, we fitted the measured repetition rate to a function of the form $y\left(t\right)=Aexp\left(-t/T_{\mathrm{cell}}\right)+mt$, which includes both an exponential decay of time constant $T_{\mathrm{cell}}$ and a linear drift of rate *m*. We found that the linear drift rate $m=\left(-4\pm 2\right)\times 10^{-15}$/day is small but non-zero. Figure 2 shows the measured frequency data, binned into daily averages and plotted over time. Additionally shown is a fitted exponential decay function with A=211 Hz and $\tau_{\mathrm{cell}}=38$(10) days, in agreement with the value predicted by Equation (2). We have offset the y-axis so that the fit and data gradually approach 0, which would correspond to the optical frequency that would be observed in the absence of any helium contamination. The right axis of the plot also converts the frequency axis into partial pressure of helium using the known frequency shift coefficients [30]. Extrapolating back in time, we infer that the helium pressure when the vacuum pumping began (approximately 60 days prior to the measurement) was 3 mTorr, very close to the atmospheric concentration of 4 mTorr.

### 3.3. Optical Clock Stability

**Figure 3:**
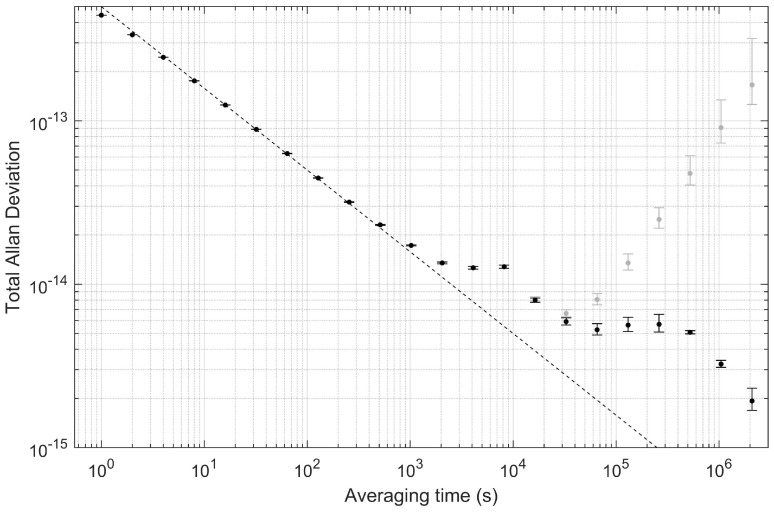
Total Allan Deviation determination of optical clock stability after removing the effect of helium by fitting and subtracting an exponential decay function with linear drift (black markers). Additionally shown in gray markers is the Total Allan deviation of the same data prior to removing the drift. At one day (86,400 s) the fractional frequency deviation is approximately $5\times 10^{-15}$. The dashed line corresponds to $5\times 10^{-13}/\tau$, with τ the averaging time in seconds.

**Figure 4:**
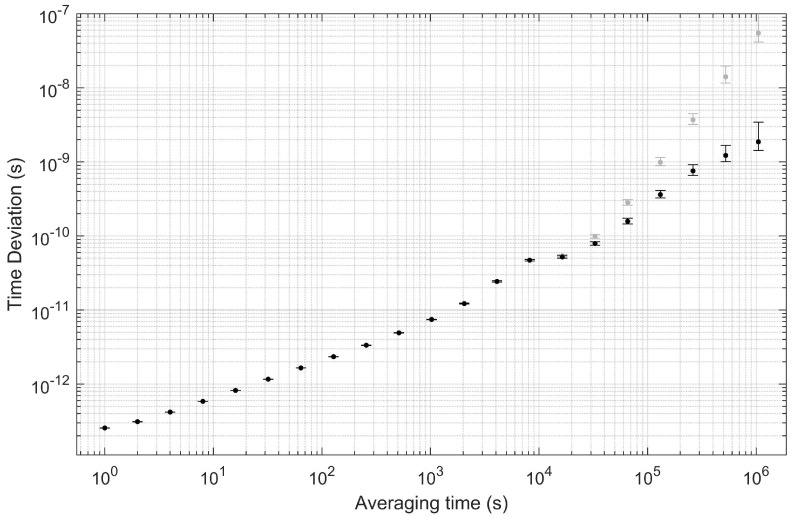
Time deviation determination of optical clock stability after removing the effect of helium by fitting and subtracting an exponential decay function (black circles). Additionally shown in gray markers is the time deviation for the same data prior to removal of the drift. At one day (86,400 s), the time deviation is approximately 200 ps.

After removing the helium-induced frequency shift, we analyze the optical clock’s stability with the Total Allan deviation [35] (Figure 3) and time deviation (Figure 4). The Total Allan deviation displays the fractional frequency deviation on the y axis against the averaging or data-binning time on the x axis, and it can be used to read off the expected amount of frequency noise occurring on any particular timescale. We observe white-frequency noise scaling as $5\times 10^{-13}/\sqrt{\tau }$, with τ the averaging time in seconds, on timescales from 1 s to 1000 s. The dashed line in Figure 3 provides a guide of how the stability would scale if the $\sqrt{\tau }$ behavior could be maintained. Instead, from $10^{3}$ s to $10^{4}$ s, the stability is flat, indicating some source of noise at around $10^{-14}$; we suspect the noise is due to small fluctuations of the laser power that are not captured by the laser power feedback servo. On longer timescales, the stability remains in the range of a few times $10^{-15}$. Note that one day corresponds to 86,400 s, at which point the Total Allan deviation is around $5\times 10^{-15}$. On the longest timescales displayed out to tens of days, the Total Allan deviation appears to reduce further, but this is possibly an artifact of the slow drift removal performed with the exponential decay function. Figure 4 displays the same data but as an accumulated time error. The two-photon clock is able to maintain a time error below 1 ns for approximately 4 days.

We searched for correlations between the observed clock frequency and various environmental monitors including lab weather and temperature sensors. We found no significant correlations. Throughout the measurement, the lab temperature was constant to within 2 ${}^{\circ }$C, and the relative humidity varied over a range of 15%.

### 3.4. Absolute Frequency

The absolute frequency of the clock can also be extracted from the frequency data, provided we account for any perturbations to the clock states experienced by the atoms. The perturbations requiring correction given the feasible accuracy of this measurement are the ac Stark shift (also known as light shift) due to the 778.1 nm laser electric field, Rb–Rb collisions within the vapor cell, the 2nd-order Doppler shift, the blackbody radiation shift due to blackbody electric fields, and the gravitational redshift. Additionally, we include in the error budget the possibility of systematic errors from servo lockpoint error and offsets in the microwave frequency chain. Effects that are negligible at the current level of uncertainty include line pulling [22] and the dc Stark shift [36].

The ac Stark shift for the two-photon clock has been calculated and measured [36] with high precision. However, without any direct attempt to measure the size of the shift in this apparatus, we must rely on an imprecise knowledge of the laser intensity to calculate the shift. With an intensity radius (1/$e^{2}$) of $w_{0}=2.1\left(3\right)$ mm and one-way laser power of 10(1) mW, we calculate a light shift of −183 Hz, and an uncertainty in this correction of 55 Hz.

Rb–Rb collisions were measured as a function of gas pressure [30], and the relevant coefficient is −13.5(10) MHz/Torr, where we are careful to specify the shift as applying to a single 778.1 nm laser that provides both photons for the spectral signal. While the saturated vapor pressure of Rb as a function of temperature is known [37], accurate temperature sensors are required to determine this pressure. Temperature sensors were placed near the designed coldest point on the vapor cell, which serves as a condenser, as well as on the main chamber of the cell. The sensors read between 98.0 and 100.0 ${}^{\circ }$C. We model this situation with a uniform distribution since we know the average temperature of the vapor must be between the hottest and coldest point; after including additional uncertainty due to sensor calibration error, this produces an expected temperature of 99(1.7) ${}^{\circ }$C, which produces a frequency shift of −2930(400) Hz.

The 2nd-order Doppler shift can be calculated in fractional frequency as ${\bar{v}}^{2}/2c^{2}$, with $\bar{v}=8k_{B}T/m\pi$ the characteristic atomic velocity and *c* the speed of light. Here, T=100 ${}^{\circ }$C is the atomic temperature, *m* the atomic mass of Rb-87, and $k_{B}$ is the Boltzmann constant. The shift is 194 Hz, and the uncertainty due to temperature sensor inaccuracy is negligible.

Next we treat the blackbody radiation Stark shift. The glass vapor cell has low transparency in the 5–20 μm window relevant to room-temperature blackbody fields. Consequently, the temperature of the vapor cell itself determines the intensity of the blackbody field. Apart from the cold spot, which is largely out of view of the vapor, the vapor cells are maintained uniformly at T=100 ${}^{\circ }$C. Previous calculations [36] find the shift at this temperature to be −195 Hz. We assign a conservative 10 % error bar to account for inaccuracies in the calculation and temperature measurement.

Clocks are known to tick slower in a gravitational field as predicted by general relativity. The calibration of the hydrogen maser by GPS corrects the hydrogen maser to the geoid, i.e., to mean sea level. Using the known coarse elevation of the laboratory in Albuquerque, NM, we assign an elevation of h=1617(5) m above sea level. The shift in fractional frequency between two clocks separated by height *h* is $gh/c^{2}$, with *g* the gravitational acceleration near earth’s surface, which we find to be 68(1) Hz.

Due to the relatively broad linewidth of the two-photon clock (typically 550 kHz as measured in our apparatus), a small error in the lockpoint can cause a significant frequency error. We searched for this effect by intentionally varying the error signal size via its dependence on the PMT high voltage, the laser modulation depth, and the laser modulation frequency. Through these trials, the size of the error signal was varied three-fold, and we observed changes in the clock frequency as large as $5\times 10^{-12}$. We therefore take the uncertainty due to servo error as 1900 Hz.

Table 1 summarizes these correction factors and their uncertainties. After applying these correction factors and maser calibration, we find the frequency of the unperturbed 5s ${}^{2}S_{1/2}\left(F=2\right)\to 5d{\;}^{2}D_{5/2}$(F = 4) transition in Rb-87 to be 385,284,566,371,190 Hz with an uncertainty of 1970 Hz. The final uncertainty includes a contribution of $1\times 10^{-13}$ fractionally due to possible inaccuracies in the hydrogen maser calibration; this contribution is negligible in the final error, which is dominated by the servo error and Rb–Rb collision shift uncertainties. This absolute measurement agrees well with prior measurements [9,10], showing a deviation of 2.8±3.9 kHz with [10]. “n/a” means “not applicable.”

## 4. Discussion

In this measurement, we have demonstrated improved long-term frequency performance of a simple two-photon optical clock. Some of the key performance metrics for deployable clocks include short-term stability (demonstrated in this work as $5\times 10^{-13}$), long-term drift rate ( $4\times 10^{-15}$/day), and long-term frequency floor (3–5 $\times 10^{-15}$). Improvements to all three can be imagined, and will depend to the ability to measure and stabilize laser power. A high laser power could be utilized to decrease the short-term stability by generating greater atomic fluorescence [22]; however, this would likely come at the expense of long-term frequency stability and drift unless the quality of the laser power stabilization is increased. Other schemes for laser power stabilization or Stark shift mitigation have been proposed and/or demonstrated [11,36,38] and may prove highly beneficial.

While work continues to improve the long-term stability of simple optical clocks such as the two-photon rubidium clock discussed here, we also note that the observed performance metrics demonstrated here already equal or surpass those of portable RF clocks [39]. A great challenge remains to miniaturize the optical clock components and produce systems that can be operated autonomously for long periods of time. Key aspects of this work will include lower-power laser sources [20], compact atomic geometries [13] with high detection efficiency, and overall reduction in electronic components and their associated size, weight, and power. With a great interest internationally amongst researchers and industry, the demonstration of a turn-key, fully portable optical clock is expected within a few years.

## Figures and Tables

**Table 1:** Summary of the frequency shifts that were corrected, and their associated uncertainties.

Effect	Frequency Shift (Hz)	Uncertainty (Hz)
ac Stark shift	−183	55
Rb–Rb collisions	−2930	400
2nd-order Doppler	194	1
Blackbody radiation	−195	19
Gravitational redshift	68	1
Servo error	0	1900
Maser calibration	n/a	39
	−3044	1970

## Data Availability

Requests for data may be sent to the corresponding author; however, restrictions apply to the availability of these data.
